# A method for determining equivalent hardening responses to approximate sheet metal viscoplasticity

**DOI:** 10.1016/j.mex.2021.101554

**Published:** 2021-10-19

**Authors:** Hamid Reza Attar, Nan Li, Alistair Foster

**Affiliations:** a Dyson School of Design Engineering, Imperial College London, London SW7 2DB, UK; b Impression Technologies Ltd, Coventry CV5 9PF, UK

**Keywords:** Sheet forming, Hot stamping, Warm forming, Viscoplasticity, Strain hardening, Design for manufacture, Design guidelines

## Abstract

Recently developed elevated temperature metal forming technologies improve material formability and address the springback issues of cold forming. However, the behaviour of alloys at elevated temperatures is viscoplastic and therefore considerably more complex than under cold forming conditions. This complex behaviour creates a barrier for industrial designers when designing for elevated temperature processes, and therefore leads to these processes often being overlooked. To overcome this barrier, a new method is proposed here to determine simpler strain hardening responses that are equivalent to the viscoplastic responses of alloys at elevated temperatures. The equivalent hardening responses are expressed by single material hardening curves and their hardening exponents are taken as parameters to approximate sheet metal viscoplasticity. This method therefore makes it possible to develop early-stage design guidelines that consider different materials and stamping processes. The method was successfully applied to two viscoplastic alloys under hot stamping conditions to determine equivalent hardening responses. The feasibility of the method has been assessed through comparing finite element simulations using the determined equivalent material models with ones using viscoplastic models. The result showed that the thinning distributions obtained were consistent in both cases, providing evidence that the method can be applied to a range of component designs.

• The proposed equivalent material models are simpler than existing viscoplastic material models and can be derived directly from experimental stress-strain data.
• Creating design guidelines from equivalent hardening exponents enables a straightforward way to compare between cold and hot stamping capabilities. This comparison makes it possible to make manufacturing process and material selection decisions quickly and effectively at the onset of a design process.
• Design guidelines enabled by the proposed method are critical resources to encourage the uptake of new elevated temperature metal forming technologies that facilitate lightweighting and improved environmental efficiency.

## Graphical abstract

**Figure:**
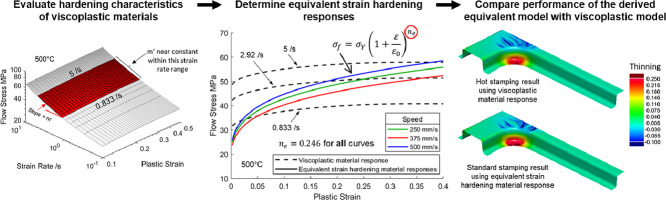

Specifications table

**Table:** 

Subject Area:	Engineering
More specific subject area:	Design for sheet metal forming processes
Method name:	Novel equivalent strain hardening method for approximating viscoplasticity
Name and reference of original method:	N/A
Resource availability:	N/A

**Method details**

## Motivation

Traditional cold stamping processes currently used in the automotive and aerospace industries are limited by poor material ductility and springback issues. To address these issues, elevated temperature processes have been invented in recent years [1,2]. One of these processes is the novel non-isothermal Hot Forming and cold die Quenching (HFQ®) process for lightweight high strength aluminium alloys developed by Lin *et al.* [1]. During this process, a blank is heated and soaked at its solution heat treatment (SHT) temperature to gain excellent formability before being stamped and quenched between cold dies to maintain a desirable tempered microstructure [3]. HFQ therefore has the potential to enable challenging geometric features, such as tight radii, to be included in component designs formed from high strength aluminium alloys. Several examples of high strength aluminium alloy components with complex design features are given by Impression Technologies Ltd [4].

However, evidence from industry discussions has revealed a key bottleneck in limiting the spread of HFQ technology as well as other hot stamping technologies [2]. It has been observed during the industrial exploitation process that there is a lack of familiarity among industrial component designers about how to apply HFQ technology during component design. This unfamiliarity arises because the material response is significantly more complicated under HFQ conditions compared with more familiar, traditional cold stamping processes. Consequently, component designers are often discouraged by the increased design overhead when adopting HFQ technology, meaning its potential cost and weight saving benefits are not fully realised.

The material response of alloys during cold forming processes is elastic-plastic and can often be described by a simple elastic-plastic material equation. One such example is given by Eq. (1), where $\sigma_{f}$ is the flow stress, $\sigma_{Y}$ is the initial yield strength, ε is the equivalent plastic strain, $\varepsilon_{0}$ is a pre-strain constant as defined by Marciniak, Duncan & Hu [5] and n is the strain hardening exponent.

(1) $$\sigma_{f}=\sigma_{Y}{\left(1+\frac{\varepsilon }{\varepsilon_{0}}\right)}^{n}$$

**Fig. 1:**
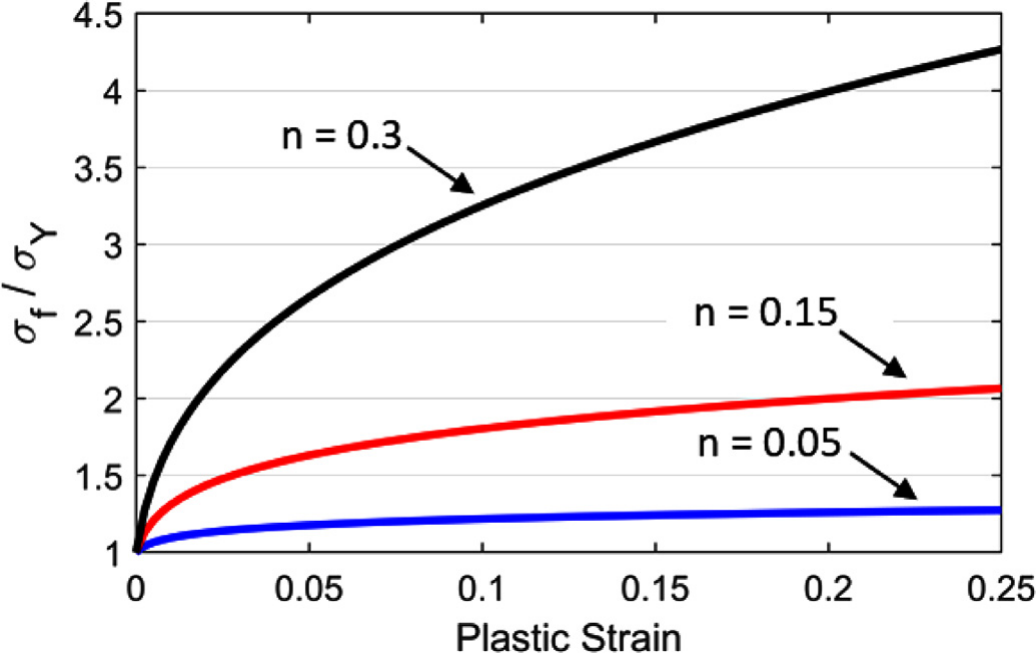
Examples of material hardening curves plotted from Eq. (1) for various n-values.

Fig. 1 illustrates three examples of hardening curves at various strain hardening exponents, denoted as n-value in this paper, generated from Eq. (1). The strain hardening exponent was chosen in this study as the parameter to represent the strain hardening characteristics; the higher this is, the greater the strain hardening. The parameters, $\varepsilon_{0}$ and $\sigma_{Y}$, were chosen as the arbitrary constants 0.002 and 80 MPa respectively, based on values provided by Marciniak, Duncan & Hu [5]. The important aspect is the strain-hardening behaviour rather than the initial yield strength $\sigma_{Y}$. The greater the strain-hardening, the better the material will perform under significant tensile deformation, as explained by Marciniak, Duncan & Hu [5]. The flow stress is normalised and plotted on the Y-axis of Fig. 1 to showcase these important strain hardening characteristics.

The simple elastic-plastic material model governed by Eq. (1) assumes a single material hardening curve with an n-value to represent the material strain hardening characteristics. However, strain hardening usually reduces at elevated temperature conditions, such as those used for the HFQ process, due to static and dynamic recovery of dislocations as described by Lin [6]. When forming aluminium alloys at elevated temperatures, viscoplastic strain rate effects on the flow stress instead become dominant.

Published literature readily addresses modelling the material viscoplastic constitutive response under HFQ conditions. For example, Mohamed *et al.* [3] and Shao *et al.* [7] developed sets of constitutive equations for modelling the viscoplastic flow of AA6082 under hot forming conditions. These viscoplastic models provide an important component of the overall modelling strategy that takes place prior to component manufacture. However, those material models are complicated, involving many parameters, because they are intended to be used in conjunction with high fidelity FE simulations of the stamping process. Such simulations demand material models that are sophisticated enough to account for softening due to damage [3], effects of biaxial strain states [7], and prediction of post-form geometry. As mentioned by Attar, Li and Foster [8] as well as Horton *et al.* [9], accurate forming simulations usually take place late in the product development process, and are not suitable for early-stage concept designers, who lack knowledge in computation and forming processes. Therefore, a simplified approach is needed to approximate the elevated temperature strain rate dependent material behaviour during early-stage design phases.

## Equivalent strain hardening response method

### Method description

**Fig. 2:**
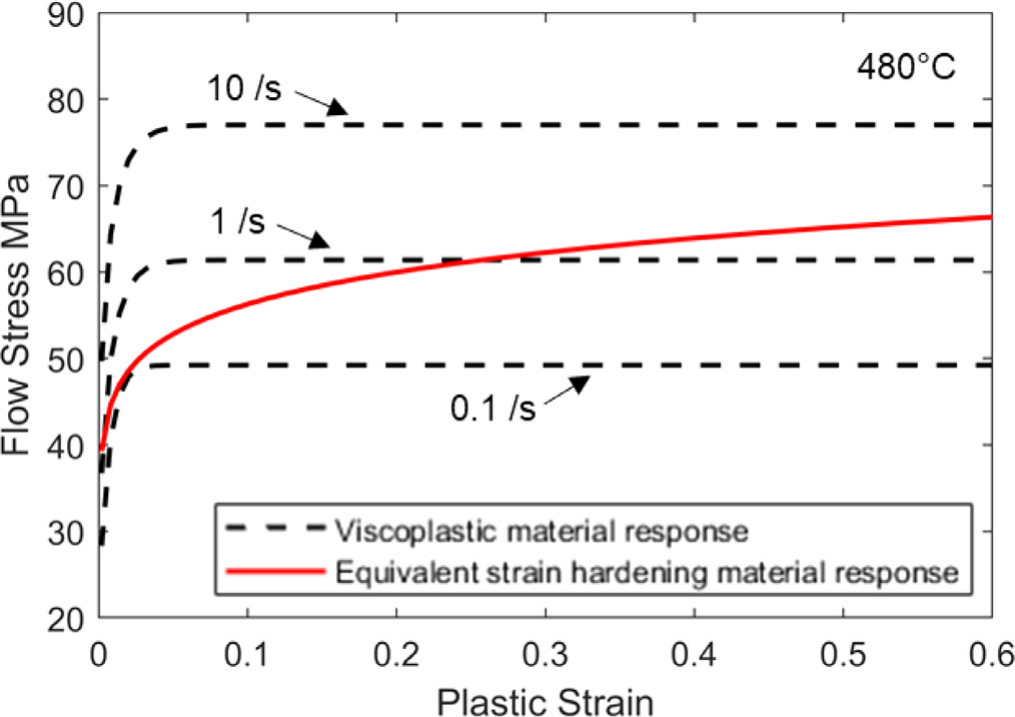
An example of an elevated temperature viscoplastic material response of AA7075 from the viscoplastic model by Zhu *et al.*[10] (black dashed lines) and its derived equivalent strain hardening response (red solid line).

It is evident that strain rate hardening has a similar effect to that of strain hardening on the forming behaviour, in terms of suppressing unstable local tensile deformation [6]. Therefore, the viscoplastic strain rate hardening characteristics can be used to derive an equivalent strain hardening elastic-plastic curve in the form of Eq. (1). The n-value of this new equivalent strain hardening curve can now be taken as an *equivalent* strain hardening exponent that represents the combined strain hardening and strain rate hardening effects on the flow stress. An example of an equivalent strain hardening curve is conceptually visualised in Fig. 2, together with the actual viscoplastic material response of AA7075 [10] that was used for its derivation. The theory for obtaining an equivalent strain hardening curve from elastic-viscoplastic material data is detailed in the following subsection.

**Fig. 3:**
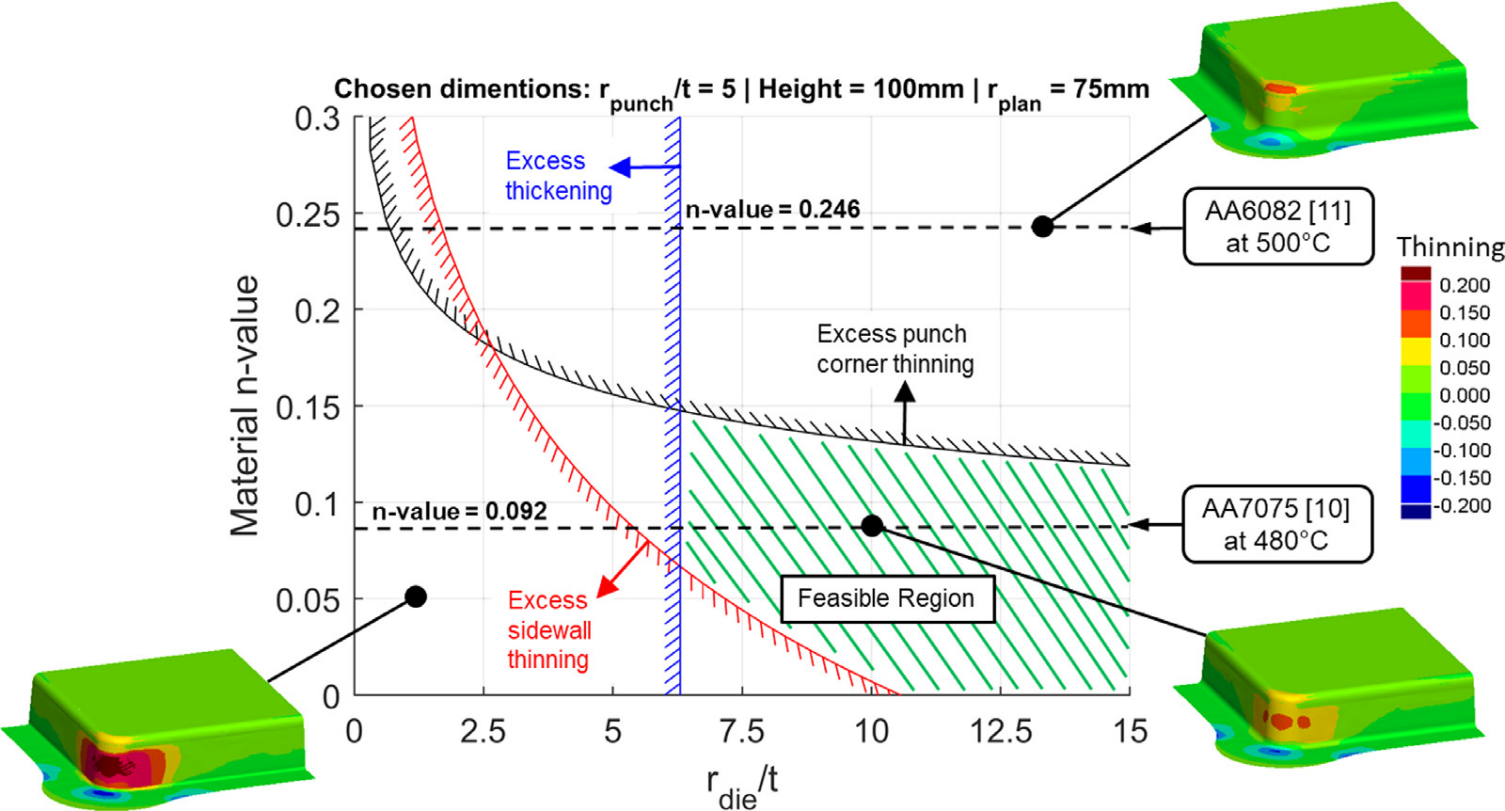
An example of a corner design map developed by Attar, Li and Foster [8], where the feasible and infeasible design regions are mapped out. The corner design map considers the proposed equivalent strain hardening exponent, denoted by Material n-value on the Y-axis. The black dashed lines correspond to equivalent strain hardening exponents proposed in this paper which were derived using the viscoplastic models of AA7075 by Zhu *et al.*[10] and AA6082 by Shao [11].

The determined equivalent n-value depends on the material stress-strain data and manufacturing conditions under which that data was obtained (e.g., cold, or hot stamping conditions). Giving consideration to the n-value during the development of early-stage component design guidelines [8,9] therefore enables material and stamping process selection decisions to be made at the onset of a design process. An example of such a design guideline is shown in Fig. 3, where the feasible and infeasible design zones are dependent on the material and stamping process that is inherent within the material n-value plotted on the Y-axis. The design guidelines enabled by the proposed method are therefore capable of rapidly assessing the likelihood of component feasibility through different stamping processes and materials, without the need for complicated FE simulations, during early design and concept phases. This in turn has the potential to increase familiarity among industrial designers and therefore reduce the barriers to adoption of new elevated temperature processes such as HFQ. For more information on the development of the design guidelines, the reader is referred to the original paper by Attar, Li and Foster [8].

Detailed FE simulations using more comprehensive viscoplastic material models can then be reserved only for designs that pass the early-stage assessments provided by the design guidelines. The role of these FE simulations would then be to verify the feasibility of detailed component designs and specify the production settings such as stamping speed and blank shape. Thus, design guidelines that use the equivalent material model developed in this study play a crucial role in guiding design decisions, while reducing the need for extensive FE simulation iterations on non-feasible concept geometry, material, and stamping process combinations.

### Simplification of HFQ forming conditions for early-stage design

Aside from strain rate dependencies, the material formed at elevated temperatures is also temperature dependent, which was characterised during the hot uniaxial tensile tests of AA6082 by Mohamed *et al.* [3]. For aluminium alloys, elevated temperatures can include warm forming (isothermal), and HFQ (non-isothermal). HFQ conditions are discussed here, which represent the most complex forming conditions [14]. In a typical HFQ process, the material is first heated to its solution heat treatment temperature (e.g., 525 °C for AA6082), then immediately transferred to the cold press and formed as outlined by Mohamed *et al.* [3]. Although the process is non-isothermal, the specified initial forming temperature is constant for a particular stamping operation and strain rate remains the only influential factor when compared to traditional cold forming processes.

Stamping speed is a process parameter which strongly influences strain rates seen during forming. For simplicity during early-stage design, it is assumed here that high stamping speeds will be used in practice. This assumption is supported by research that has shown the benefits of forming components under HFQ conditions at high stamping speeds. High speeds take advantage of strain rate hardening, increase post form strength potential by utilising fast quenching [3], and reduce the time taken for undesirable flange cooling to occur during material drawing. As a result, El Fakir *et al.* [12] saw improved thickness homogeneity when forming a wing stiffener component under HFQ conditions at 500 mm/s compared to 250 mm/s. Conventional presses typically operate at speeds of up to 500 mm/s in modern industrial settings as stated by Adam, Brazier & Foster [13]. In addition, HFQ is not practical for many complex geometries at speeds below 250 mm/s [12]. Therefore, high speeds within this range are considered in this study.

Given that high stamping speeds will be used for HFQ in practice, it is further approximated here that cooling effects during material drawing have negligible influence on the thinning distribution when considered in the context of a design guideline. Therefore, the stamping conditions of HFQ are approximated to those of elevated temperature isothermal processes. Fine tuning of stamping speeds, along with other process parameters, is assumed to take place at the late FE modelling stage of product development. This fine tuning stage will be conducted by HFQ process experts using modern (and often proprietary) state-of-the-art simulation methods, as mentioned by Attar, Li and Foster [8].

Following these approximations made for early-stage design purposes, a new method to simplify viscoplastic material characteristics at elevated temperatures into an equivalent strain hardening response is proposed in this section. The theoretical basis for the method is now outlined.

### Theoretical basis

**Fig. 4:**
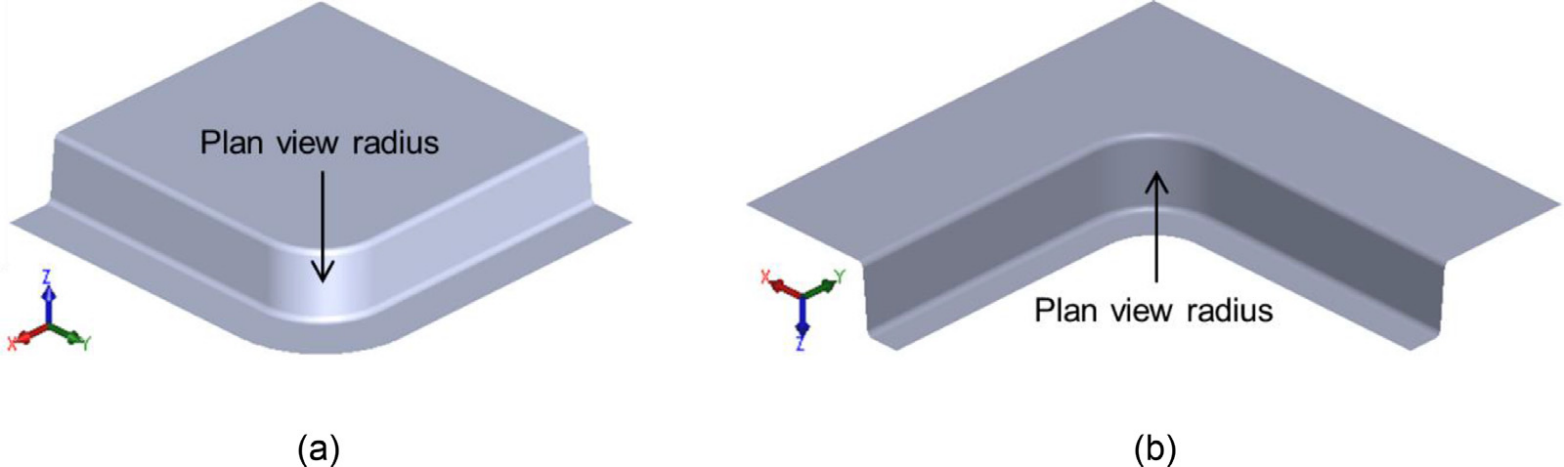
(a) shrink corners and (b) stretch corners.

To facilitate the development of the equivalent strain hardening exponent method, the forming responses of corner geometries were considered. Corner geometries are common limiting design features, found in a wide variety of automotive components, and can be broadly classified into two categories: shrink corners, and stretch corners, as shown in Fig. 4. Shrink corners are formed when material is drawn under a circumferential compression into a plan view radius which is smaller than the local blank radius. Representative examples of components with shrink corners are automotive door inners and electric vehicle battery boxes. Conversely, stretch corners form when material is drawn under a circumferential tension into a plan view radius which is larger than the local blank radius, and are found on B-Pillar components or similar.

Isothermal forming simulations of both corner types were first conducted to analyse the forming strain versus time response. Quarter deep drawn box geometries were considered for shrink corners and half B-Pillar geometries were considered for stretch corners. Symmetry boundary conditions were used in all FE simulations. The elastic-viscoplastic material model published by Zhu *et al.* [10] was adopted and implemented into the commercial FE simulation software PAM-STAMP to simulate the viscoplastic behaviour of AA7075 at elevated temperatures. Both the blank and the tools were kept at a forming temperature of 480°C [10].

**Fig. 5:**
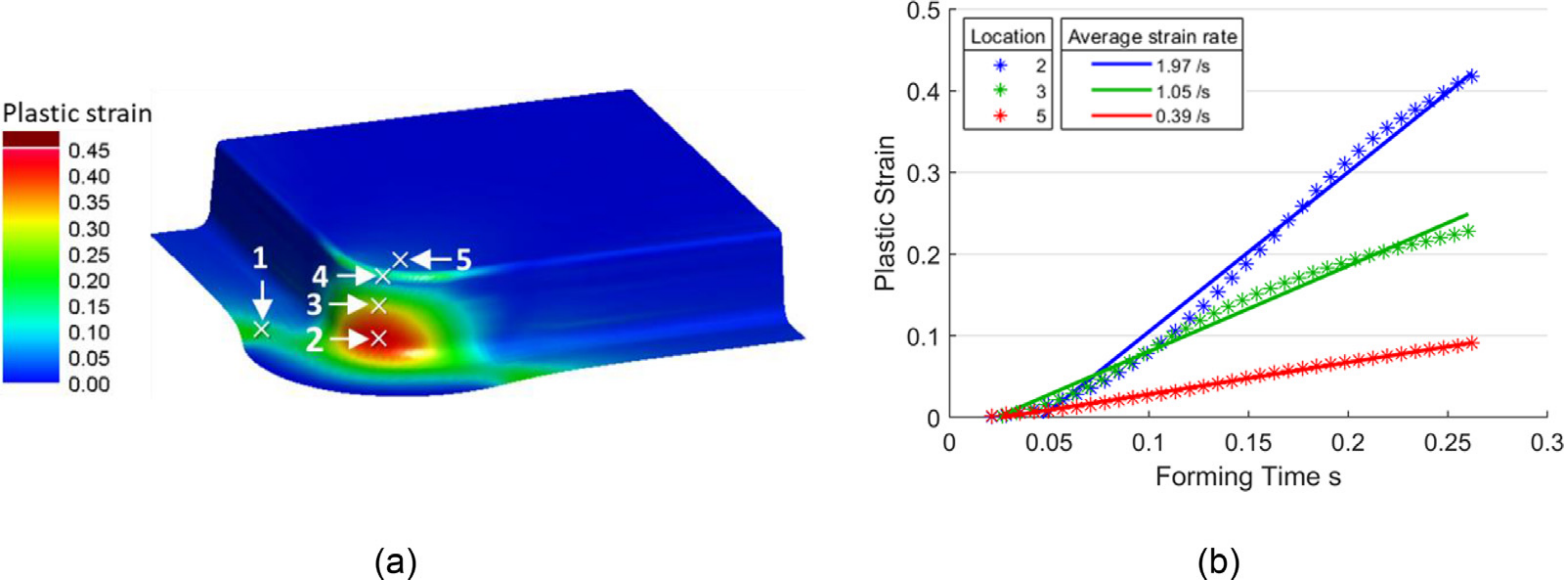
Shrink corner response: (a) Plastic strain field and representative data collection locations and (b) plastic strains approximately proportional to the forming time; data is obtained at 375 mm/s under isothermal conditions at 480°C using the AA7075 material model by Zhu *et al.*[10].

Fig. 5(a) shows a typical equivalent plastic strain field, hereafter referred to as plastic strain field, from an as-formed arbitrary deep drawn box geometry of height 100 mm. Five representative locations covering various strain levels, as shown in the figure, were selected to examine the plastic strain histories. It was found that the plastic strains extracted from all locations increased near proportionally with forming time. Three representative strain histories are shown in Fig. 5(b) for clarity. Consistent near-linear strain histories were found after checking a wide range of deep drawn box geometries. The strain histories are approximately linear because the stamping speed was constant, and necking did not occur. These observations lead to constant increases in compressive strains at the shrink corner flange area during material drawing. The constant increases in compressive strains resulted in continual increases in resistance to material flow during stamping, as explained by Attar, Li and Foster [8]. It is acknowledged that the strain histories are not perfectly linear since material drawing occurs as well as stretching. Nevertheless, the linear engineering approximation was considered as reasonable, and it enabled the following equations to be derived. Based on the near-linear trendlines in Fig. 5(b), an average strain rate can be defined for shrink corners as in Eq. (2)

(2) $$\dot{\varepsilon }=\frac{d\varepsilon }{dt}≅\varepsilon \frac{S}{H}$$

where ε and $\dot{\varepsilon }$ are the plastic strain and average strain rate respectively, t is the forming time, H is the drawing depth and S is the stamping speed.

**Fig. 6:**
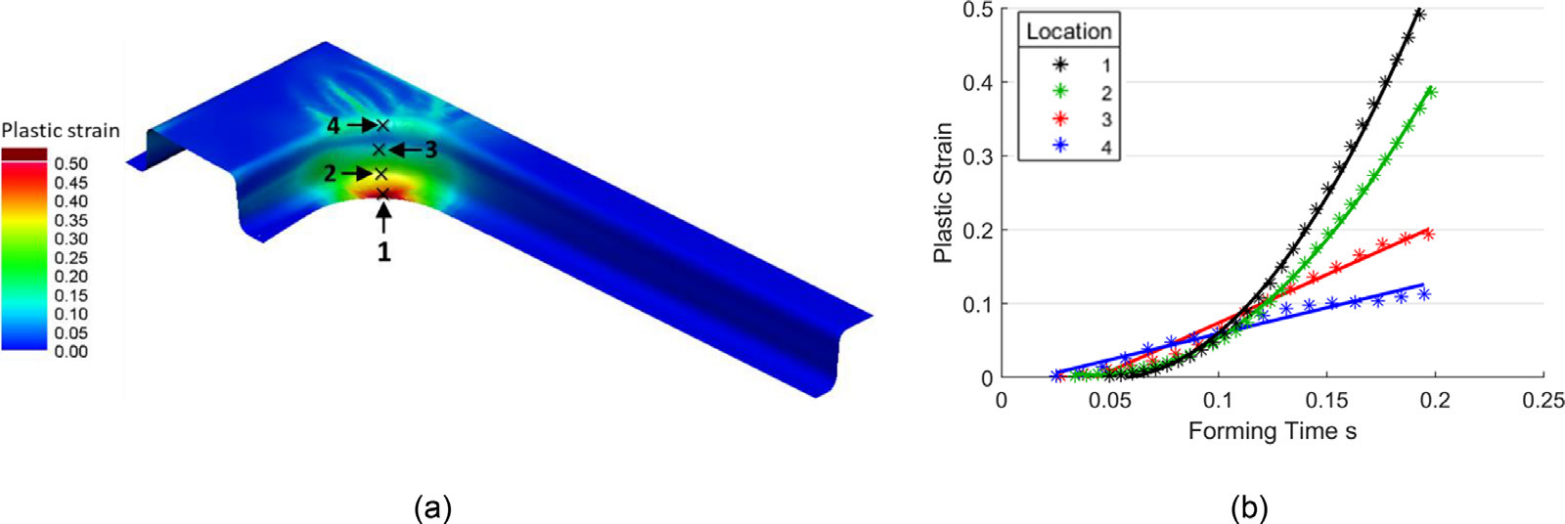
Stretch corner response: (a) Plastic strain field and representative data collection locations and (b) latter stage plastic strains approximately proportional to the forming time; data is obtained at 250 mm/s under isothermal conditions at 480°C using the AA7075 material model by Zhu *et al.*[10].

Eq. (2) was obtained by considering the strain response of shrink corners. The same analysis is now repeated for stretch corners. Fig. 6(a) shows a typical strain field from an as-formed arbitrary B-Pillar geometry of height 50 mm. Four representative locations covering various strain levels, as shown in the figure, were selected to examine the plastic strain histories that are plotted in Fig. 6(b). In contrast to the box geometry, the plastic strain histories near the critical zone of maximum plastic strain (i.e., locations 1 and 2) were found to be non-linear for the B-Pillar. Approximately linear strain histories were found at less critical zones (i.e., locations 3 and 4) with lower plastic strains.

**Fig. 7:**
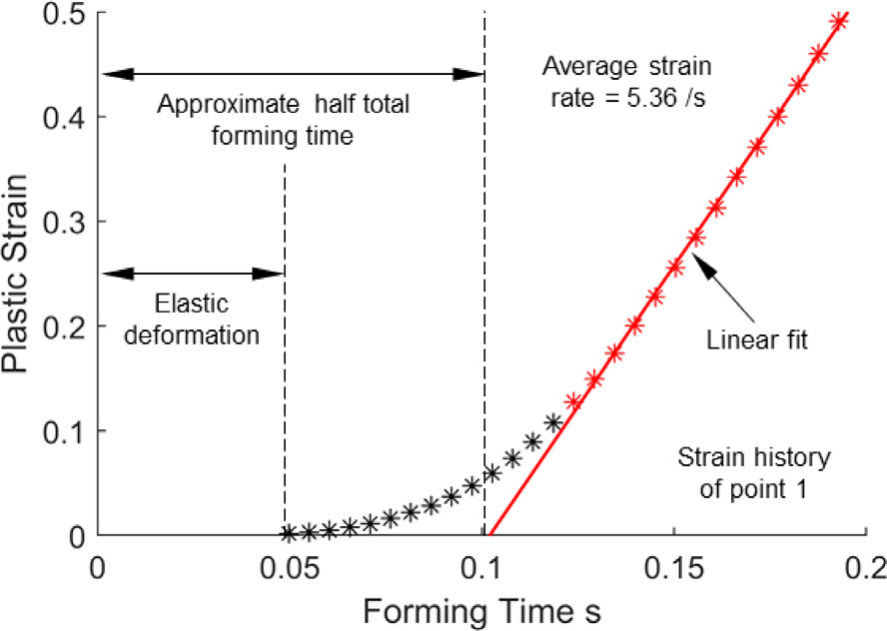
Strain history of point 1 in Fig. 6 considering half the forming time to obtain a linear fit.

The strain history extracted from point 1 in Fig. 6 is replotted in Fig. 7 where the non-linear trend with the points can be observed. Most of the strain was induced into the material at approximately the latter half of the forming time, and the material was under elastic deformation for the first quarter. Therefore, to approximate a linear strain history for the B-Pillar component with a stretch corner, only half the total forming time was considered, where a good linear fit was obtained. Treating the strain histories as linear is a key approximation to aid in the following analysis. The proposed method is therefore most valid for geometries with similar material flow characteristics to the corners seen in this study, or geometries with near-linear plastic strain history over some fraction of the total forming time. Based on the linear trendlines in Fig. 6(b) over half the total forming time for the high plastic strain zones, an average strain rate for stretch corners can be approximated as in Eq. (3), where there is a factor of 2 difference with Eq. (2).

(3) $$\dot{\varepsilon }=\frac{d\varepsilon }{dt}≅\varepsilon \frac{2S}{H}$$

Taking the log with base 10 of Eq. (2) for shrink corners gives Eq. (4).

(4) $$\log\dot{\varepsilon }=\log\varepsilon +\log S-\log H$$

**Fig. 8:**
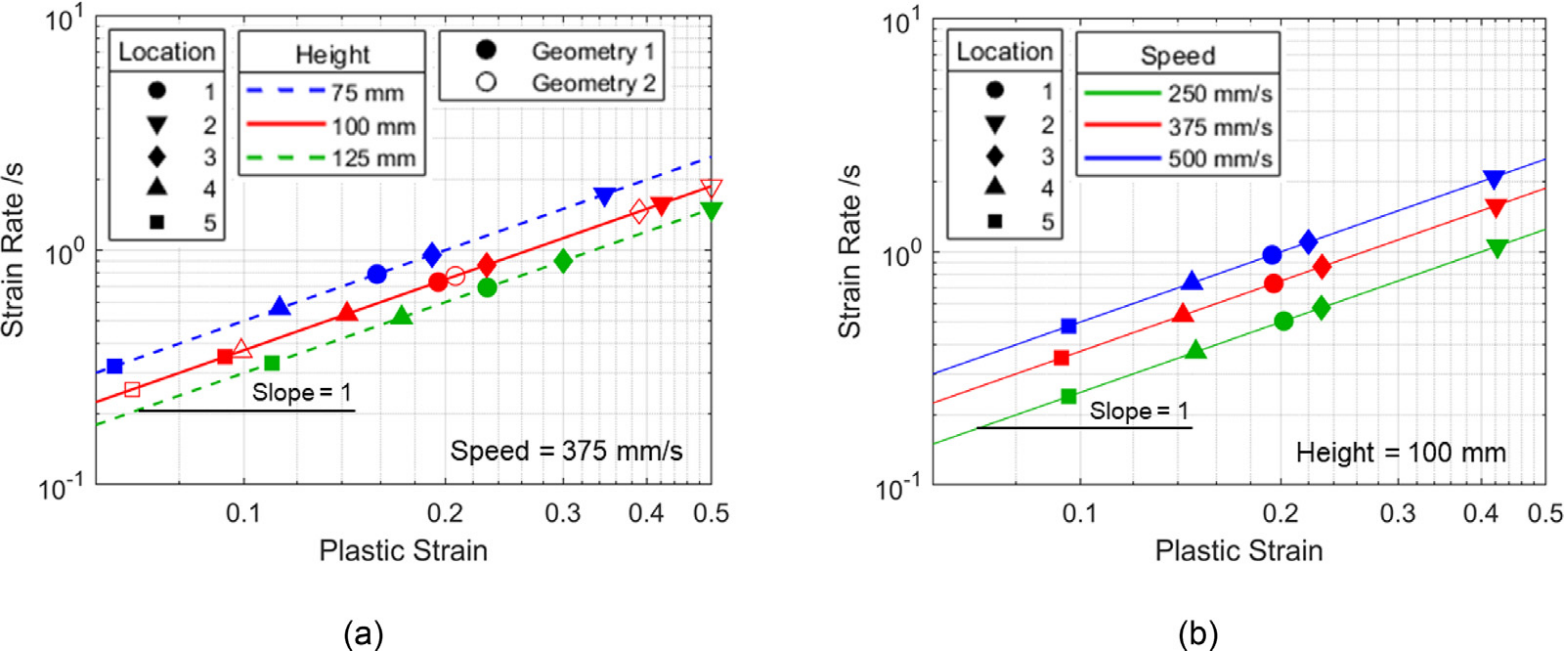
Average strain rates and plastic strains from various locations based on final stage simulation data as in Fig. 5(a), shown to be parallel in logarithmic scales when varying (a) geometry and (b) forming speed.

It can be seen from Eq. (4) that the relationship between plastic strain and average strain rate is linear with a slope of 1 in logarithmic scales. The points plotted in Fig. 8 are strain - average strain rate abscissa-ordinate pairs that were obtained from the locations considered in Fig. 5(a) for various geometries and stamping speeds. The fitted lines are based on Eq. (4), where it confirms that the trendlines remain parallel in logarithmic scales under all scenarios. Geometries 1 and 2 denoted in Fig. 8(a) represent two arbitrary corner fillet radii combinations, where plastic strain values differ but still lie on the same trendline. To summarise, the gradients of these trendlines are independent of the component design and stamping speed; the intercepts are independent of the corner radii combinations but dependent on the corner height (i.e., drawing depth) and stamping speed, as shown in Fig. 8(a) and (b) respectively.

**Fig. 9:**
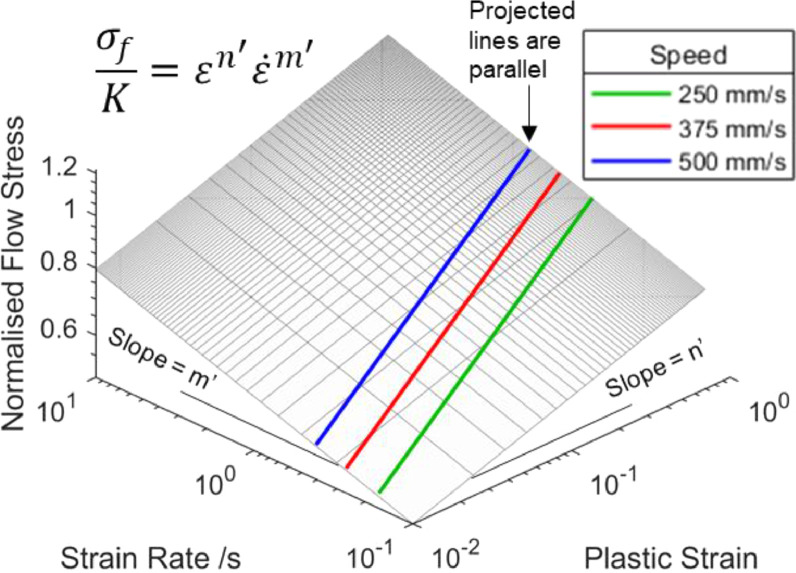
Flat stress surface plotted from Eq. (5) in logarithmic scales. The flat surface demonstrates the constant $n^{\prime }$ and $m^{\prime }$ slopes. The coloured lines are projections of the lines from Fig. 8(b) onto the stress surface.

Next, by assuming a viscoplastic material follows a power law constitutive relationship [6] defined by Eq. (5)

(5) $$\sigma_{f}=K\varepsilon^{n^{\prime }}{\dot{\varepsilon }}^{m^{\prime }}$$

where $\sigma_{f}$ denotes flow stress, the governing function of the flow stress in logarithmic scales can be rewritten as

(6) $$\log\sigma_{f}=\log K+n^{\prime }\log\varepsilon +m^{\prime }\log\dot{\varepsilon }$$

where K is the strength coefficient, and $n^{\prime }$ and $m^{\prime }$ are the strain and strain rate hardening exponents respectively. It can be seen from Eq. (6) that the relationship between flow stress, strain and strain rate is linear in logarithmic scales, and the slopes take the values of the corresponding hardening exponents, defined by Eqs. (7) and (8) respectively. The quantities $n^{\prime }$ and $m^{\prime }$ can be obtained from experimental stress-strain data. To demonstrate the constant $n^{\prime }$ and $m^{\prime }$ slopes in logarithmic scales, a graphical representation of the flat stress surface is plotted in Fig. 9.

(7) $$\frac{\partial \ln\sigma_{f}}{\partial \ln\varepsilon }=\frac{\partial \log\sigma_{f}}{\partial \log\varepsilon }=n^{\prime }$$

(8) $$\frac{\partial \ln\sigma_{f}}{\partial \ln\dot{\varepsilon }}=\frac{\partial \log\sigma_{f}}{\partial \log\dot{\varepsilon }}=m^{\prime }$$

Since both $n^{\prime }$ and $m^{\prime }$ are constants, the stress surface obtained from Eq. (5) is flat in logarithmic scales. Consequently, the parallel lines in the strain – strain rate plane in Fig. 8 remain parallel when projected to the flat stress surface, as illustrated in Fig. 9 for three stamping speeds. The flow stress – strain curves should satisfy both Eqs. (4) and (6). By substituting Eq. (4) into (6), Eq. (9) can be obtained. This equation represents the projection of the flow stress curve to the flow stress – plastic strain plane in logarithmic scales.

(9) $$\log\sigma_{f}=\left(n^{\prime }+m^{\prime }\right)\log\varepsilon +\log\left(K{\left(\frac{S}{H}\right)}^{m^{\prime }}\right)$$

It can be seen from Eq. (9) that the stamping speed and height (i.e., draw depth) only influence the flow stress curve offset, and do not change its slope, graphically illustrated by the parallel lines in Fig. 9. Accordingly, a total strain hardening exponent $n_{total}$ can be defined as in Eq. (10), by differentiating Eq. (9) with respect to logε. This total strain hardening exponent combines the strain hardening and strain rate hardening material characteristics.

(10) $$n_{total}=\frac{d\log\sigma_{f}}{d\log\varepsilon }=n^{\prime }+m^{\prime }$$

Substituting Eq. (2) and Eq. (10) into Eq. (5) results in Eq. (11), which describes an equivalent strain hardening response based on the combined strain and strain rate hardening material characteristics, where $C_{1}$ is a material and forming time dependent constant.

(11) $$\sigma_{f}=K\varepsilon^{n^{\text{'}}}{\left(\varepsilon \cdot \frac{S}{H}\right)}^{m^{\text{'}}}=K{\left(\frac{S}{H}\right)}^{m^{\text{'}}}\varepsilon^{n^{\text{'}}}\varepsilon^{m^{\text{'}}}=C_{1}\varepsilon^{n_{\mathrm{total}}}\;\text{where}\;C_{1}=\;K{\left(\frac{S}{H}\right)}^{m^{\text{'}}}$$

If the same analysis is repeated for stretch corners, (i.e., by using Eq. (3) instead of Eq. (2)), then $C_{1}$ should be replaced with $C_{2}$, and then Eq. (11) still holds for stretch corners.

(12) $$C_{2}=\;K{\left(\frac{2S}{H}\right)}^{m^{\prime }}$$

This is because it was shown empirically in Fig. 6(b) that most of the strain in the high strain area occurred over half the total forming time. This observation resulted in a factor of 2 difference between Eq. (3) for stretch corners and Eq. (2) for shrink corners. It is noteworthy that the type of corner considered (i.e., shrink or stretch) does not influence the total strain hardening exponent, but only influences the scaling factors $C_{1}$ and $C_{2}$. The total strain hardening exponent can therefore be taken as a simple material parameter to describe the combined strain and strain rate hardening effects, detailed further below. Implementation of this method on two grades of viscoplastic aluminium alloys is now discussed.

### Theory implementation on viscoplastic materials

**Fig. 10:**
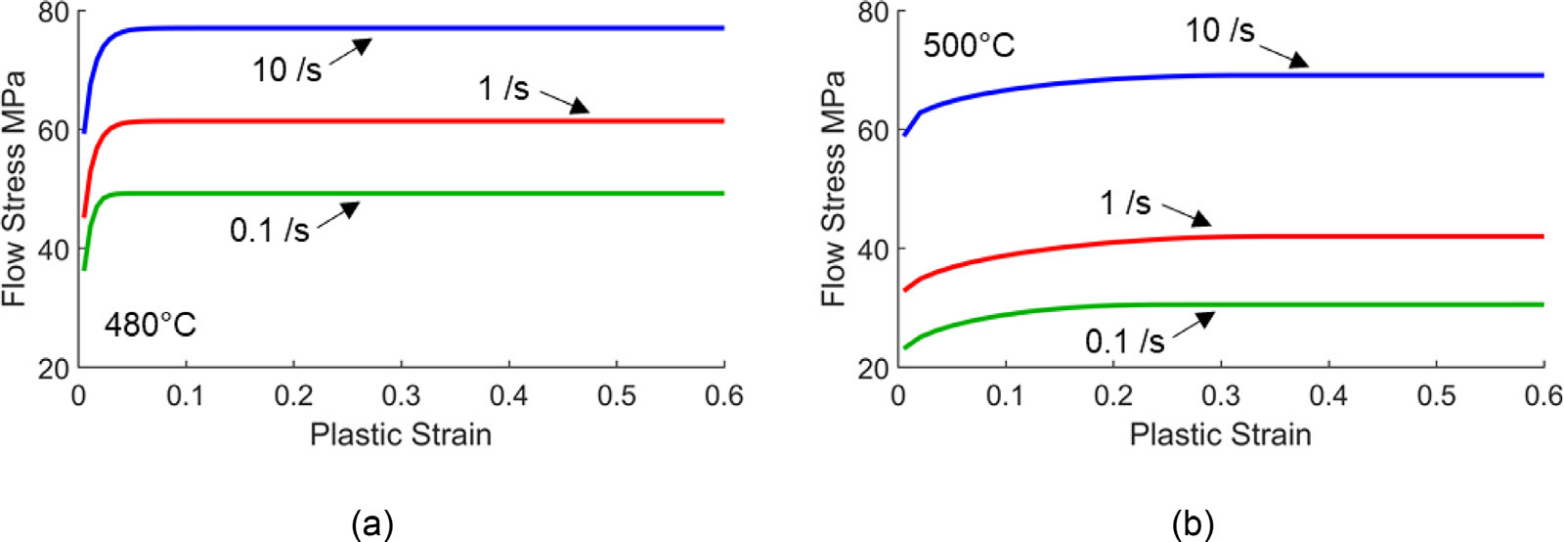
Flow stress - plastic strain curves from two viscoplastic material models: (a) AA7075 at 480 °C by Zhu *et al.*[10] and (b) AA6082 at 500 °C by Shao [11].

The theoretical basis introduced above is now implemented on two viscoplastic material models from the literature. The material models considered are for AA7075 by Zhu *et al.* [10] at a forming temperature of 480 °C and for AA6082 by Shao [11] at 500 °C. The flow stress curves are plotted in Fig. 10 from these material models. The viscoplastic responses of these aluminium alloys at elevated temperatures can be observed by the increase of flow stress with strain rate. These models were chosen for demonstration due to their different hardening characteristics, as described in more detail in the following subsections. It should be noted that experimental stress – strain data could also be used in the following analysis.

When the strain and strain rate hardening exponents $n^{\prime }$ and $m^{\prime }$ of a real material are constant, the power law flow behaviour based on Eq. (5) can be assumed. Under these conditions, it was seen that a total hardening exponent could be defined in Eq. (10) for forming deep corners that was independent of component design and stamping speed. The $n^{\prime }$ and $m^{\prime }$ values for the viscoplastic material models considered in Fig. 10 are now analysed to understand to what extent the power law flow rule could be approximated for real materials.

#### Viscoplastic material model for AA7075 by Zhu et al. [10]

**Fig. 11:**
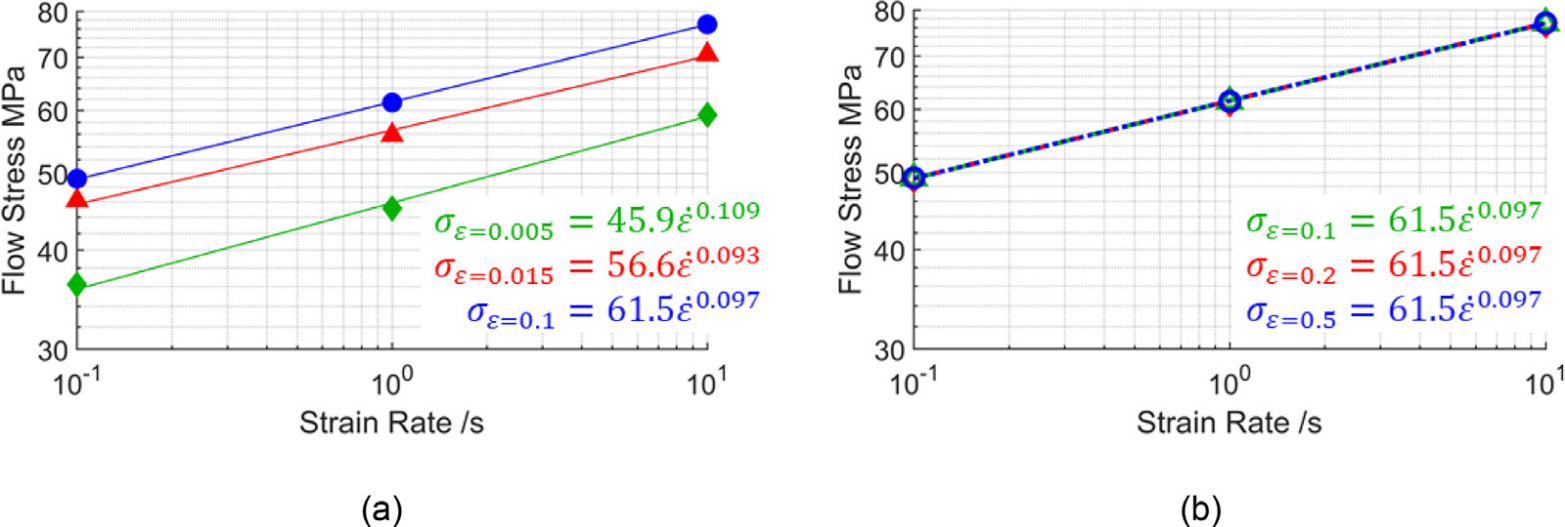
Flow stress against strain rates of 0.1 /s, 1 /s and 10 /s in logarithmic scales at different strain levels (a) 0.005, 0.05 and 0.1, and (b) 0.1, 0.2 and 0.5 calculated from the viscoplastic model for AA7075 by Zhu *et al.*[10] at 480°C.

The viscoplastic model for AA7075 by Zhu *et al.* [10] was first analysed. Good linear fits were obtained by plotting flow stress against strain rate in logarithmic scales for two strain ranges, as seen in Fig. 11. These linear fits in logarithmic scales suggest power law viscoplastic responses are present within these strain rate ranges. Further, the strain rate exponents seen from the equations in Fig. 11 are near constant, especially for the higher strain range (Fig. 11(b)). Within this higher strain range, Fig. 10(a) shows flat curves, suggesting an $n^{\prime }$-value of zero, which is confirmed by the coincident lines plotted in Fig. 11(b).

**Fig. 12:**
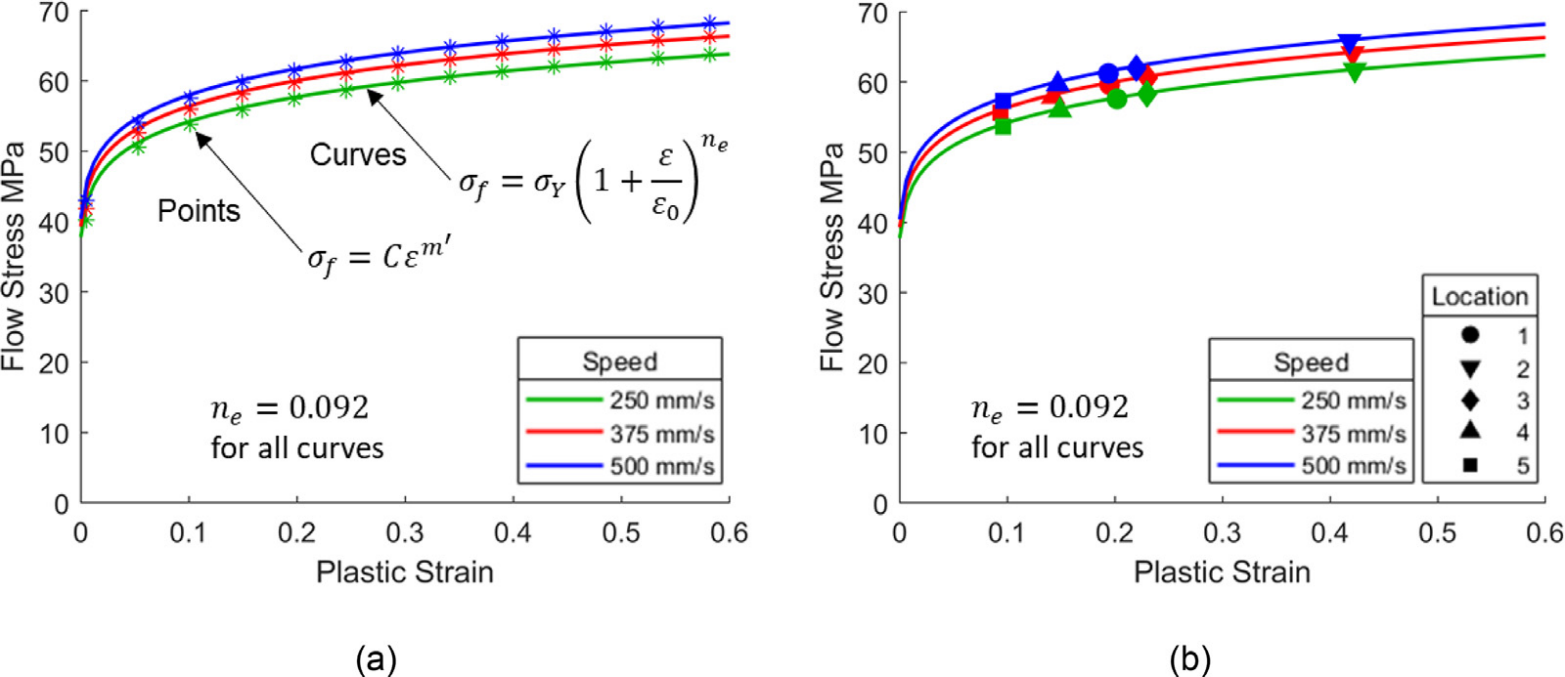
Definition of equivalent strain hardening curves for the viscoplastic model for AA7075 by Zhu *et al.*[10] at 480°C. The Fig. shows (a) fitting points from Eq. (11) to Eq. (1) and (b) the fitted equivalent curves plotted together with symbols. The symbols represent the flow stress values calculated using the viscoplastic model for AA7075 by Zhu *et al.*[10] at the locations illustrated in Fig. 5(a).

An equivalent stress response for AA7075 can then be calculated using Eq. (11). Here, $n^{\prime }=0$, meaning $n_{total}=\;m^{\prime }$ according to Eq. (10). The values of K and $m^{\prime }$ are taken as 61.5 and 0.097 respectively from Fig. 11(b), since these are at the most important strain ranges. As well as material constants, the parameter C in Eq. (11) also depends on forming time, which is governed by geometry height and stamping speed. Plotted in Fig. 12 for demonstration are stress-strain curves at three speeds and at a fixed height of 100 mm from Eq. (11). The increase of flow stress with increased stamping speed is attributed to the strain rate hardening of the material. Plotting points from Eq. (11) and refitting those points to Eq. (1) results in the definition of an equivalent strain hardening exponent $n_{e}$, shown in Fig. 12(a). All curves have the same $n_{e}$ value of 0.092 because $m^{\prime }$ is constant and therefore $n_{e}$ is independent on forming rate, as seen in the earlier analysis.

To validate the equivalent curves, flow stress values were calculated directly using the viscoplastic material model based on strains and average strain rates from the representative locations shown in Fig. 5(a). These are shown by the symbols in Fig. 12(b), plotted on top of the equivalent curves. A good agreement can be seen between the symbols and the derived equivalent curves for all speeds.

#### Viscoplastic material model for AA6082 by Shao [11]

**Fig. 13:**
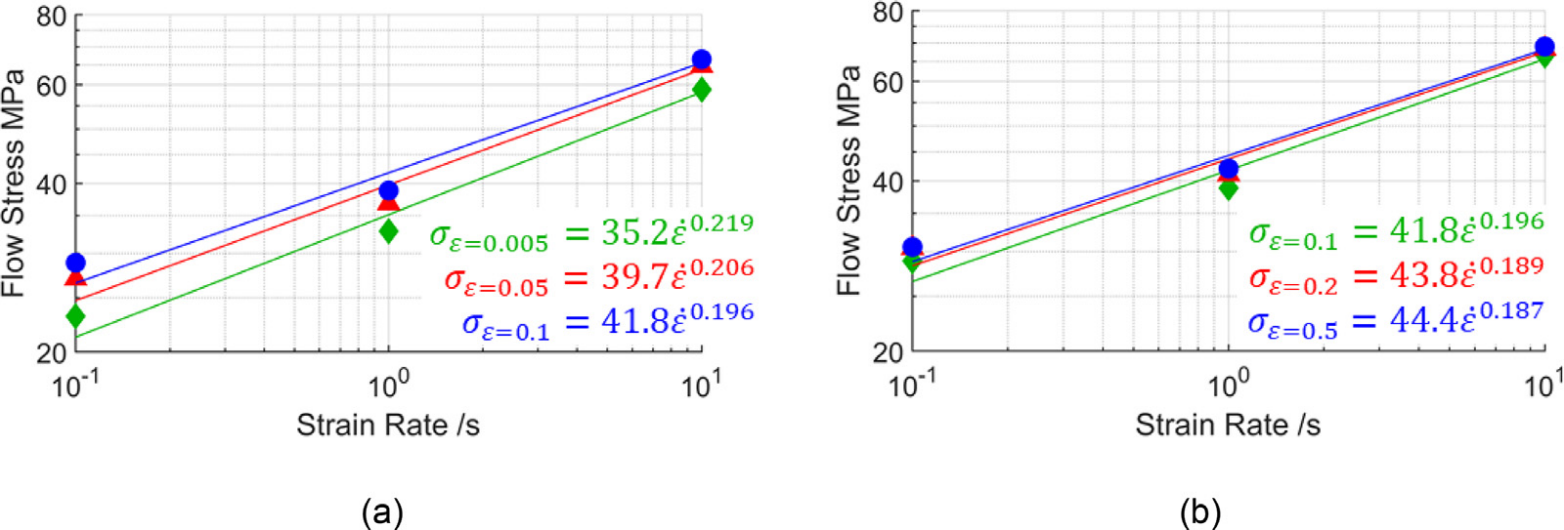
Flow stress against strain rates of 0.1 /s, 1 /s and 10 /s in logarithmic scales at different strain levels (a) 0.005, 0.05 and 0.1, and (b) 0.1, 0.2 and 0.5 calculated from the viscoplastic model for AA6082 by Shao [11] at 500°C.

The viscoplastic model for AA6082 by Shao [11] was next analysed. This time poor linear fits were seen when plotting flow stress against strain rate in logarithmic scales for strain rate ranges between 0.1 /s and 10 /s, as seen in Fig. 13. These poor linear fits in logarithmic scales suggest a non-constant $m^{\prime }$ when varying strain rate.

To facilitate power law approximations, the strain rate ranges under consideration were limited to a range such that the $m^{\prime }$ value can be considered as near constant. Eq. (2) was used to determine the strain rate range. A maximum allowable strain was defined to be the tensile strain value of AA6082 at necking, seen experimentally by Shao [11] to be approximately 0.5 at 500 °C. Along with this maximum strain, two extreme forming scenarios (i.e., speeds and heights) were defined, which allowed the strain rate limits to be calculated based on Eq. (2):

1. Speed of 500 mm/s (fast), height of 50 mm (shallow): $\dot{\varepsilon }=0.5\;\times \left(\frac{500}{50}\right)=5\;/s$
2. Speed of 250 mm/s (slow), height of 150 mm (deep): $\dot{\varepsilon }=0.5\;\times \left(\frac{250}{150}\right)=0.833\;/s$

**Fig. 14:**
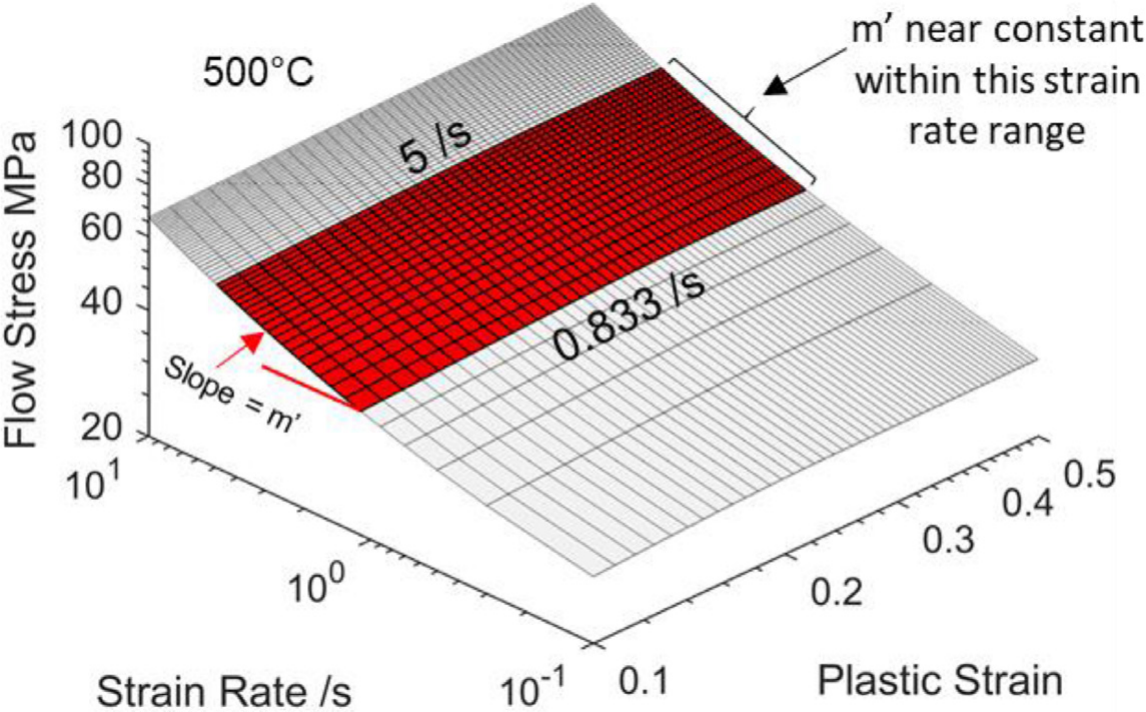
Flow stress surface at 500°C plotted in logarithmic scales from the viscoplastic material model for AA6082 by Shao [11]. The red zone bounded by strain rates of 5 /s and 0.833 /s is where $m^{\prime }$ is taken as near constant.

**Fig. 15:**
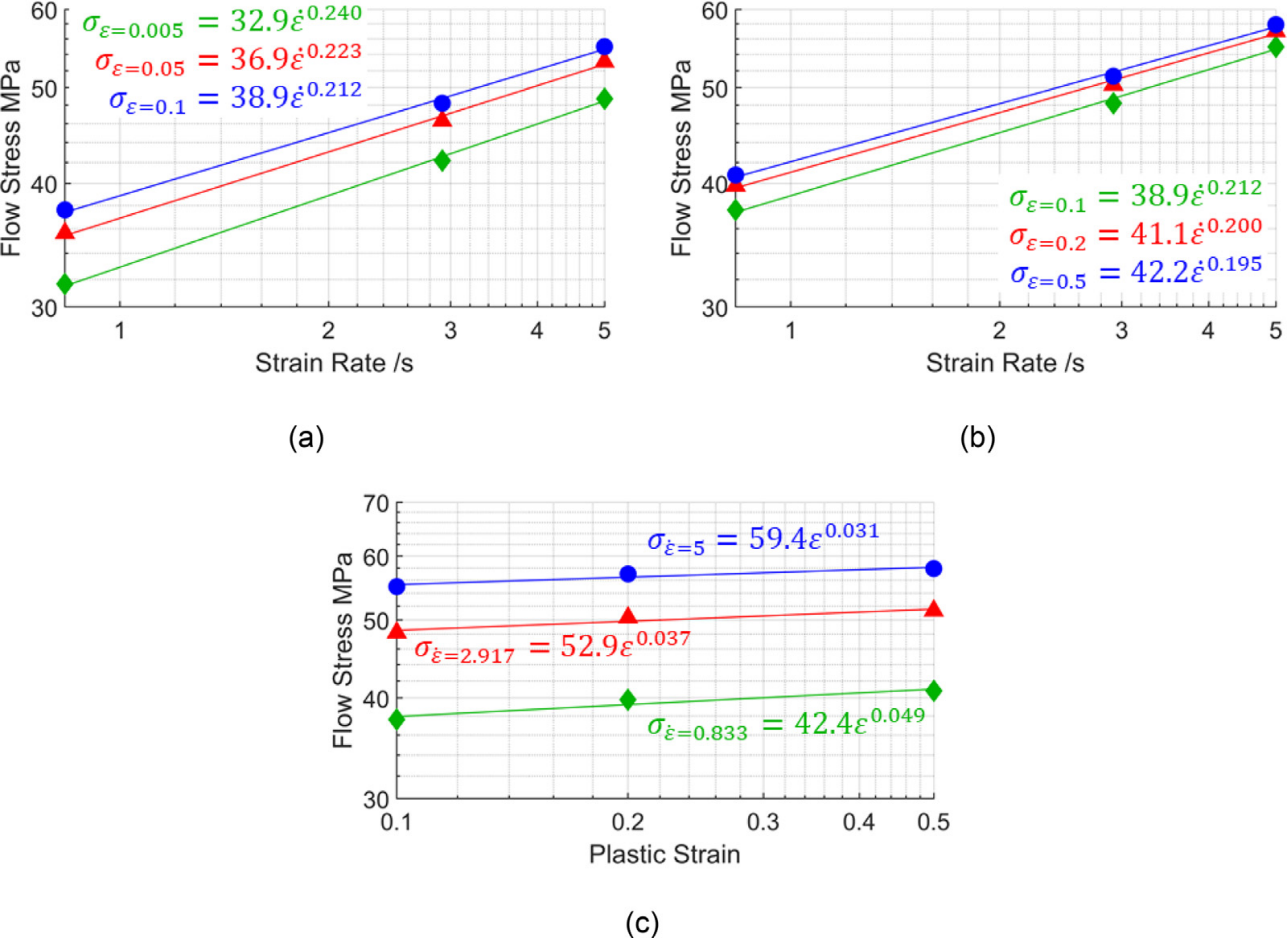
Flow stress against strain rates of 0.833 /s, 2.917 /s and 5 /s at different strain levels (a) 0.005, 0.05 and 0.1, and (b) 0.1, 0.2 and 0.5, and (c) flow stress against the higher strain range for the same three strain rates. All data is in logarithmic scales and generated using the viscoplastic model for AA6082 by Shao [11] at 500°C.

Fig. 14 illustrates the strain rate range in which the $m^{\prime }$-values are taken as near constant for the viscoplastic model proposed by Shao [11]. Plotting flow stress against strain rate within these ranges reveals good linear fits in logarithmic scales, as seen in Fig. 15(a) and (b). These good linear fits in logarithmic scales now suggest power law viscoplastic responses can be approximated within the limited strain rate ranges. Plotting flow stress against the higher strain range for the same three strain rates also shows a good linear fit in logarithmic scales in Fig. 15(c). Unlike the material model for AA7075 by Zhu *et al.* [10], the $n^{\prime }$ can no longer be taken as zero, as can be seen by the power law exponents in Fig. 15(c).

**Fig. 16:**
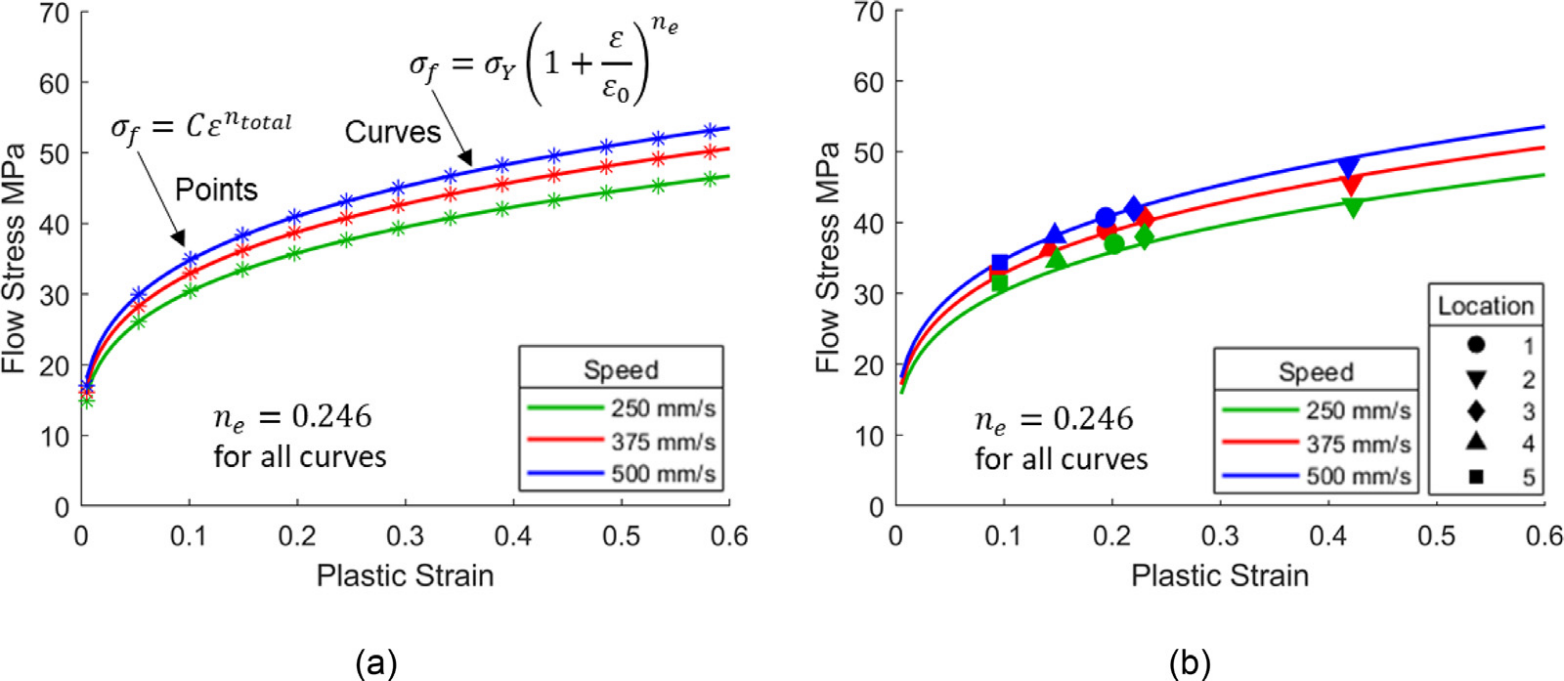
Definition of equivalent strain hardening curves for the viscoplastic model published by Shao [11] at 500°C. The Fig. shows (a) fitting points from Eq. (11) to Eq. (1) and (b) the fitted equivalent curves plotted together with symbols. The symbols represent flow stress values calculated using the viscoplastic model for AA6082 by Shao [11] at the locations illustrated in Fig. 5(a).

Within these strain and strain rate ranges of interest, near constant exponents can be seen. Averaging exponents Fig. 15(b) and Fig. 15(c) across all three strains and strain rates in the figures results in 0.202 and 0.039 for $m^{\prime }$ and $n^{\prime }$ values respectively. As before, using these $m^{\prime }$ and $n^{\prime }$ values in conjunction with Eq. (11) to generate points, then refitting those points with Eq. (1), results in the equivalent n-value $n_{e}$ of 0.246 for Shao's [11] viscoplastic material model at 500 °C. The higher $n_{e}$-value for Shao's [11] model for AA6082, when compared with the material model for AA7075 by Zhu *et al.* [10] $\left(n_{e}=0.092\right)$, can be attributed by the greater strain and strain rate hardening characteristics which were seen in Fig. 10. The equivalent curves are plotted in Fig. 16 for three speeds, where all the curves have the same $n_{e}$ value of 0.246. The symbols in Fig. 16(b) represent flow stress values calculated using the viscoplastic material model by Shao [11] based on strains and average strain rates from the locations shown in Fig. 5(a). A good agreement between the calculated symbols and derived equivalent curves can be seen.

## Method validation

The performance of the proposed equivalent material models was assessed through FE simulations. Various stretch corner and shrink corner geometries were simulated. Comparisons were made between simulations using the equivalent models with ones using viscoplastic models from the cited literature. The simulations using the viscoplastic models were performed under both isothermal and non-isothermal conditions.

### Comparison with isothermal conditions

Elevated temperature isothermal forming simulations were first conducted using the real viscoplastic material models. Both the blank and the tools were kept at a forming temperatures of 480°C and 500°C for AA7075 and AA6082 respectively. Standard forming simulations were conducted using the equivalent material models. PAM-STAMP software was used for all FE simulations, following the set-up outlined by Attar, Li and Foster [8].

**Fig. 17:**
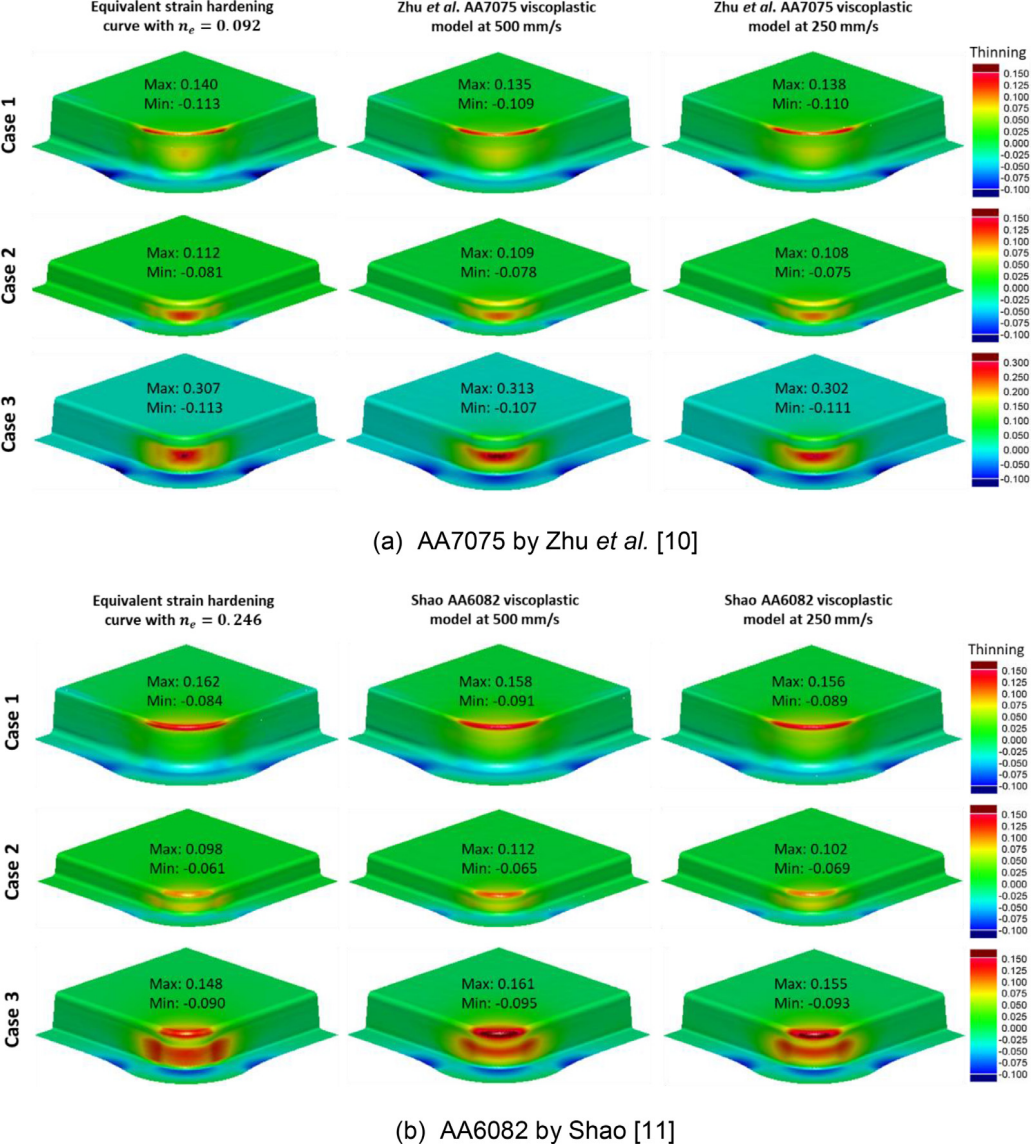
Comparison of thinning distributions on deep drawn boxes with varying geometries (Case 1, Case 2, Case 3) from simulations using the equivalent material model (cold forming) and simulations using the viscoplastic models (elevated temperature isothermal forming) at speeds 500 mm/s and 250 mm/s for (a) AA7075 by Zhu *et al.*[10] and (b) AA6082 by Shao [11].

**Fig. 18:**
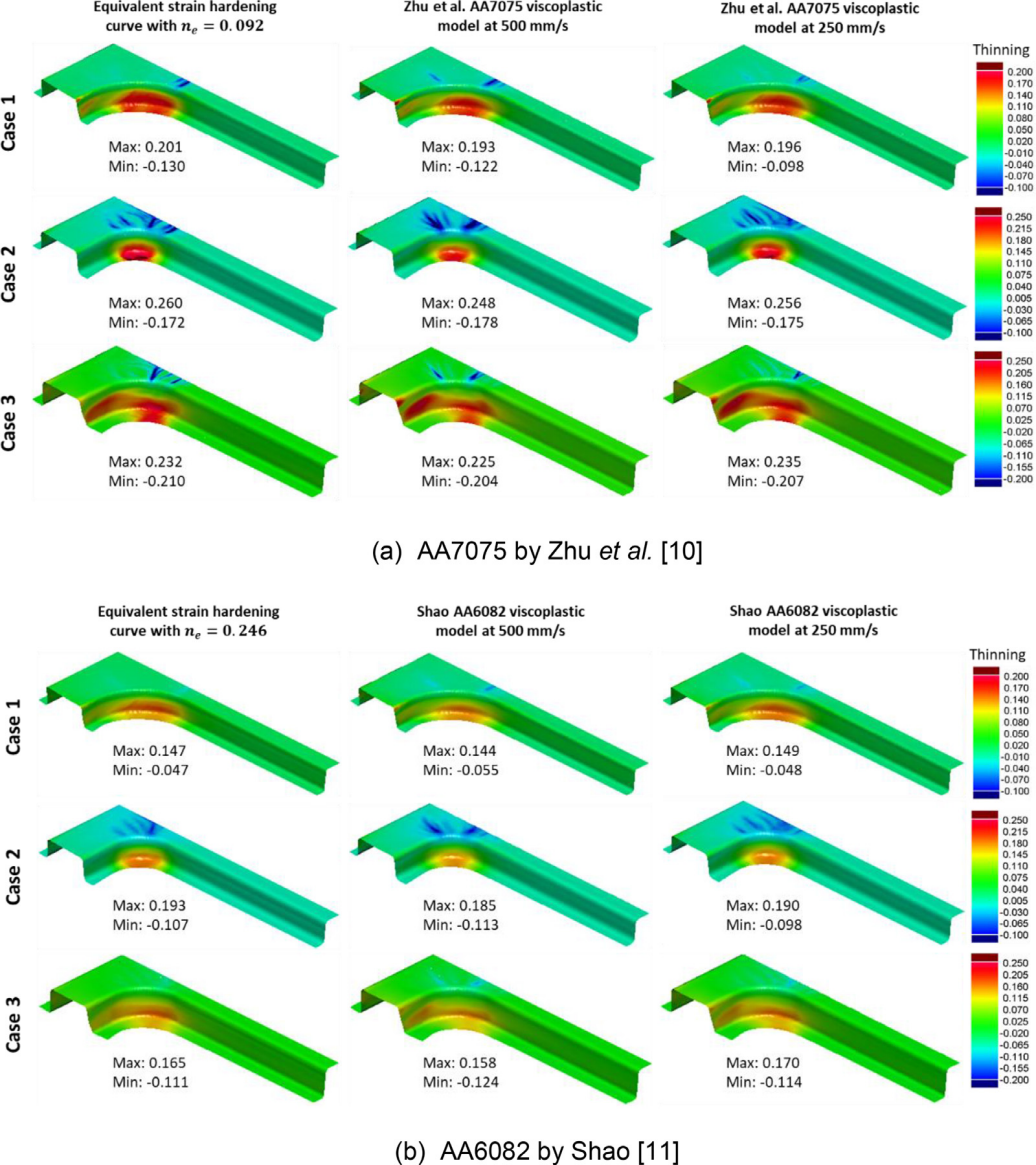
Comparison of thinning distributions on B-Pillars with varying geometries (Case 1, Case 2, Case 3) from simulations using the equivalent material model (cold forming) and simulations using the viscoplastic models (elevated temperature isothermal forming) at speeds 500 mm/s and 250 mm/s for (a) AA7075 by Zhu *et al.*[10] and (b) AA6082 by Shao [11].

Post form thinning distributions were compared, since these largely dictate the component feasibility in industrial settings [8,9]. Figs. 17 and 18 compare thinning distributions on deep drawn box geometries and B-Pillar geometries respectively. Under the elevated temperature isothermal conditions, viscoplastic material behaviour was active, and forming speeds of 250 mm/s and 500 mm/s were considered. Similar thinning distributions when varying geometries (i.e., Cases 1, 2, and 3) were seen between the viscoplastic results and their equivalent material model counterparts for all cases.

### Comparison with non-isothermal conditions

Comparisons with non-isothermal simulations were performed next. These comparisons were to determine to what extent the equivalent material models could be used to approximate the forming response in non-isothermal environments, such as those under HFQ conditions [3]. As mentioned previously, high stamping speeds are assumed to be utilised by process experts at the late prototyping phase of product development, and HFQ is not feasible at speeds below 250 mm/s [13]. Therefore, speeds of 250 mm/s and 500 mm/s were considered.

**Fig. 19:**
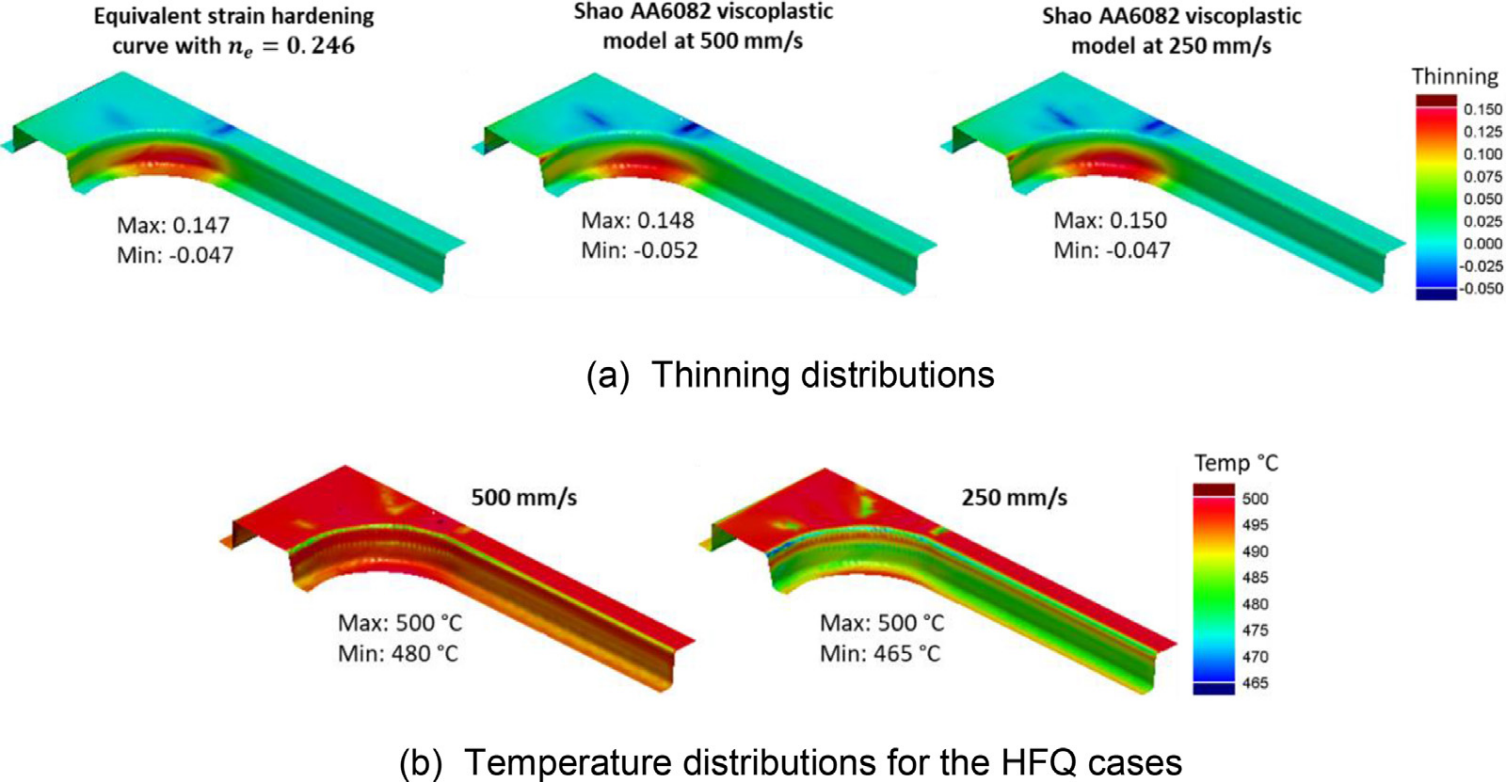
(a) Thinning distributions on a B-Pillar geometry formed from AA6082. Results obtained using the equivalent material models (cold forming) as well as using the viscoplastic models under HFQ conditions. (b) A comparison of temperature distributions for the HFQ cases. HFQ cases were formed at an initial forming temperature of 500 °C and speeds 500 mm/s and 250 mm/s.

Fig. 19(a) compares the thinning distributions of a B-Pillar geometry formed using the two different material models for AA6082 at 500 °C. Non-isothermal HFQ conditions at speeds of 250 mm/s and 500 mm/s were used for the viscoplastic material simulations. Standard forming simulations were conducted using the equivalent material model with an equivalent n-value of 0.246, as derived earlier for AA6082. The height of this B-Pillar geometry was 50 mm, as formed by Ganapathy *et al*. [2] through a non-isothermal hot stamping process. Under these conditions, forming using the equivalent material model gave a similar thinning distribution to those formed under non-isothermal HFQ conditions. Fig. 19(b) compares the temperature distributions for the two HFQ cases. A maximum temperature drop of 35 °C can be seen for the 250 mm/s case. The cooler zones were mostly situated at the fillet radii and sidewall regions, and the temperature loss in these locations was not enough to significantly influence changes in thinning for this geometry.

**Fig. 20:**
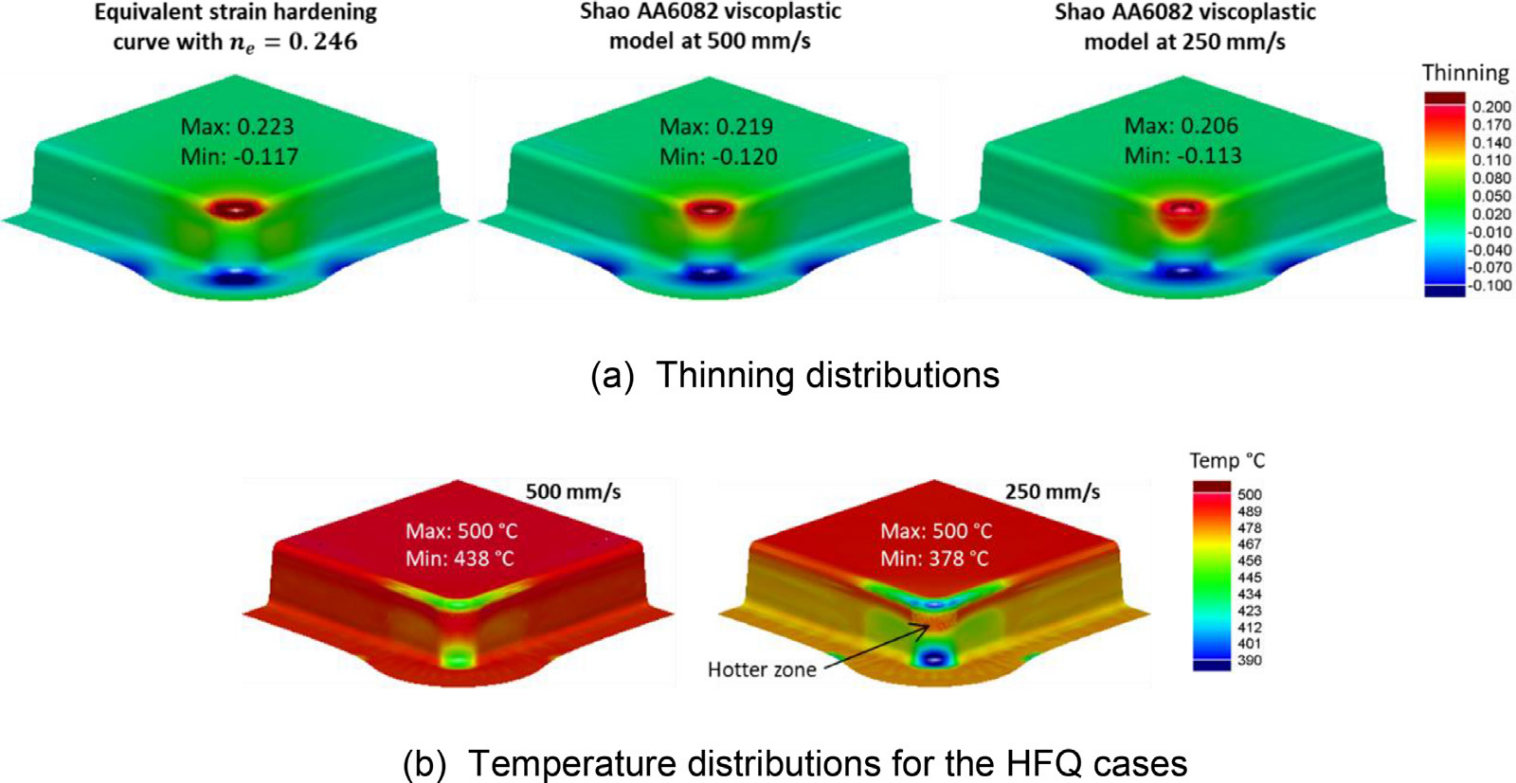
(a) Thinning distributions on a deep drawn box geometry formed from AA6082. Results obtained using the equivalent material models (cold forming) as well as using the viscoplastic models under HFQ conditions. (b) A comparison of temperature distributions for the HFQ cases. HFQ cases were formed at an initial forming temperature of 500°C and speeds 500 mm/s and 250 mm/s.

Fig. 20(a) compares the thinning distributions of a B-Pillar geometry formed using the two different material models for AA6082 at 500 °C, with the same settings as those in Fig. 19(a). Forming using the equivalent material model gave a similar thinning distribution to those forming under HFQ conditions. However, the magnitude of thinning was slightly less at 250 mm/s and the thinning was distributed more towards the component sidewall.

The height of this deep drawn box geometry was 125 mm, which is significantly higher than the B-Pillar case in Fig. 19 (50 mm). Due to the greater height, the forming time was longer, which allowed more time for heat transfer to occur during forming, particularly at the lower speed. Fig. 20(b) compares the temperature distributions for the two HFQ cases. A maximum temperature drop of 122 °C can be seen for the 250 mm/s case. Importantly, this temperature drop occurred at the zone where the material is under a circumferential compression and is already difficult to draw in [8]. Further, a relatively hotter zone was found at the sidewall, where the material is locally lower in strength [3]. This locally hotter zone resulted in a spread of thinning to the sidewall at this zone and caused the FE result at 250 mm/s to slightly deviate from the result using the equivalent material model. Nevertheless, the sidewall thinning fundamentally stemmed from the large top corner thinning zone, which in this case was due to an excessively tight plan view radius, as shown by Attar, Li and Foster [8]. This concentrated thinning zone, which was caused by improper component design, can indeed be identified during early-stage design. This early identification is enabled by using the design guidelines by Attar, Li and Foster [8] together with the equivalent material n-value, as was demonstrated in Fig. 3.

**Fig. 21:**
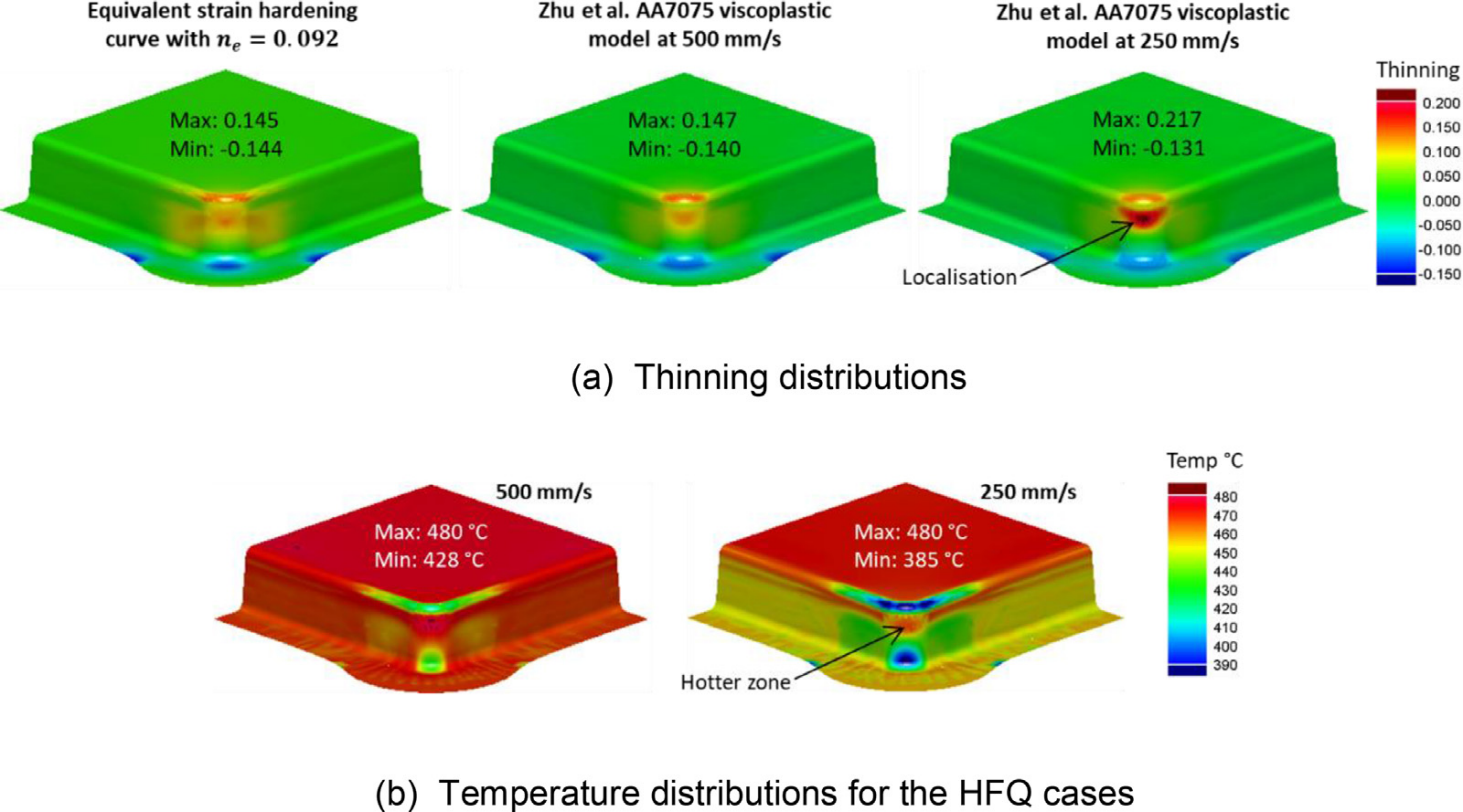
(a) Thinning distributions on a deep drawn box geometry formed from AA7075. Results obtained using the equivalent material model (cold forming) as well as using the viscoplastic models under HFQ conditions. (b) A comparison of temperature distributions for the HFQ cases. HFQ cases were formed at an initial forming temperature of 480 °C and speeds 500 mm/s and 250 mm/s.

Another noteworthy factor which may cause predictions from the equivalent material models to be less conservative in non-isothermal environments would be the material hardening characteristics. Fig. 21 shows the same geometry presented in Fig. 20, but formed from AA7075. It was shown earlier that the equivalent n-value $n_{e}$ of AA7075 material model by Zhu *et al.* [10] is 0.092, which is lower than the n-value of AA6082 by Shao [11] ($n_{e}$ = 0.246). In Fig. 21(a), thinning localisation is seen when the deep drawn box is formed at 250 mm/s for the same reasons as previously discussed. However, since AA7075 had a lower $n_{e}$ value than AA6082, the localisation was more pronounced, and caused a greater deviation from the result using the equivalent model. Note that $n_{e}$ is used here to quantitively describe the combined effects of strain and strain rate hardening. There is a deviation in the thinning distributions between the two material models seen in Fig. 21(a) despite the temperature distributions seen in Fig. 21(b) and Fig. 20(b) being alike. Nevertheless, the thinning result when forming at 500 mm/s is like that of using the equivalent model. This suggests that the equivalent model can still give a reasonable approximation of the forming response under hot stamping conditions. Therefore, the material $n_{e}$ value together with the design guidelines by Attar, Li and Foster [8] can be used to guide early-stage design for materials with lower levels of equivalent hardening.

## Summary

A method to determine an equivalent strain hardening material model to approximate sheet metal viscoplasticity was proposed. The equivalent strain hardening exponent combined strain hardening and strain rate hardening characteristics into a single material parameter. To derive the equivalent strain hardening model, linear strain histories across a fraction of the total forming time were approximated, and average strain rates were calculated. Based on the average strain rates, a new equation for the equivalent strain hardening response was derived. Implementations of the method on viscoplastic material models for AA7075 and AA6082 from the literature were presented, and equivalent hardening exponents for each material were determined. The method was validated by comparing mechanical forming simulations that used the derived equivalent strain hardening models with thermo-mechanical simulations that used viscoplastic material models. The results showed consistently similar thinning distributions between both cases for a range of component geometries.

The proposed strain hardening exponents can be taken as simple material parameters that enable the development of early-stage design guidelines. Using these design guidelines, feasible equivalent hardening exponents for candidate component designs can be determined. Therefore, the proposed method enables early-stage decision making on materials and stamping processes (e.g., cold, or hot stamping conditions) to be made quickly and effectively.

## Declaration of Competing Interest

The authors declare that they have no known competing financial interests or personal relationships that could have appeared to influence the work reported in this paper.
